# Lessons from an evaluation of an antimicrobial resistance laboratory capacity telementoring program in Ethiopia and Kenya

**DOI:** 10.1017/ash.2023.404

**Published:** 2023-09-29

**Authors:** Kieran Hartsough, Jacqueline Safstrom, Martin Evans, Manise Pierre, Edward Schroder, Carolyn Herzig, Ana Da Costa, Susan Githii, Rajiha Abubeker, Gebrie Alebachew, Surafel Fentaw Dinku, Abera Abdeta, Estifanos Tsige, Maritza Urrego

## Abstract

**Background:** Antimicrobial resistance (AMR) presents a global health threat. Training laboratory technicians to accurately identify and report AMR is critical in low- and middle-income countries (LMICs) to control the spread of AMR. Ethiopia and Kenya implemented a telementoring program, ECHO AMR, via the Project ECHO learning platform to improve laboratory technician capacity to isolate, identify, and report AMR organisms; to perform antimicrobial susceptibility testing (AST); and to develop a community of learning. Between January 2018 and January 2022, biweekly 1-hour sessions were held for 8 and 22 laboratories averaging 19 or 43 participants per session in Ethiopia and Kenya, respectively. Each session included a lecture, a laboratory challenge case presentation, and discussion. An evaluation was conducted to assess perceived strengths and weaknesses of the program and its usefulness in improving bacteriology capacity. **Methods:** In July–August 2022, semistructured key informant interviews of purposively and randomly selected laboratorians were conducted to understand participant perspectives of ECHO AMR, including session structure and content, changes in laboratory performance, and the virtual learning platform. Eligible participants attended at least one-third of available sessions in Ethiopia (8 of 26 sessions) or Kenya (5 of 16 sessions) during 2021. Key informant interviews were transcribed and systematically reviewed to identify key themes. **Results:** In total, 22 laboratory technicians participated in the key informant interviews: 12 in Ethiopia and 10 in Kenya. Participants reported that the ECHO AMR session structure was well organized but recommended increasing session duration to allow more time for discussion. Technical content was presented at an appropriate level and was highly rated. However, participants suggested including more subject-matter experts to provide the lectures. All participants reported positive change in laboratory practice, including implementation of international standards for AST, better quality control, improved confidence and critical thinking, and increased AMR awareness and reporting. Participants learned well in the virtual environment, with the platform providing wide-ranging geographic interactions to share skills and knowledge among sites without travel. However, there were connectivity issues, competing work priorities during sessions, and a lack of dedicated space for team participation. **Conclusions:** Laboratory technicians reported that virtual laboratory training was well-received, efficient, and impactful. Participants benefited both individually and collectively, as a laboratory. Suggested improvements included increasing session duration, connectivity support, and including more subject-matter experts to broaden technical content. Further assessment is needed to evaluate the ECHO AMR’s impact on laboratory practices through observation and laboratory data. Virtual programs, requiring less time and resources than traditional in-country trainings, can be optimized and used to share and increase bacteriology knowledge in LMICs.

**Disclosures:** None

